# The Effect of Dynamic Recrystallization on Monotonic and Cyclic Behaviour of Al-Cu-Mg Alloy

**DOI:** 10.3390/ma11060874

**Published:** 2018-05-23

**Authors:** Adam Tomczyk, Andrzej Seweryn, Małgorzata Grądzka-Dahlke

**Affiliations:** Faculty of Mechanical Engineering, Białystok University of Technology, Wiejska 45C Str., 15-351 Białystok, Poland; a.seweryn@pb.edu.pl (A.S.); m.dahlke@pb.edu.pl (M.G.-D.)

**Keywords:** dynamic recrystallization, mechanical behaviour, creep pre-deformation, fatigue life

## Abstract

The paper presents an investigation that was conducted to determine the possibility of the occurrence of the process of dynamic recrystallization in 2024 alloy during monotonic tensile and creep tests at the elevated temperatures of 100 °C, 200 °C, and 300 °C. As-extruded material was subjected to creep process with constant force at elevated temperatures, until two varying degrees of deformation were reached. After cooling at ambient temperature, the pre-deformed material was subjected to monotonic and fatigue tests as well as metallographic analysis. The process of dynamic recrystallization was determined in monotonic tests to occur at low strain rate (0.0015/s) only at the temperature of 300 °C. However, in the creep tests, this process occurred with varying efficiency, both during creep at 200 °C and 300 °C. Dynamic recrystallization was indicated to have a significant influence on the monotonic and cyclic properties of the material.

## 1. Introduction

EN AW-2024 aluminium alloy is one of the most typically and frequently used of the entire Al-Cu-Mg alloy group. This alloy is frequently applied whenever a beneficial ratio of strength to weight is required. Frequently, these applications are connected with the aircraft industry (e.g., plating of wings or fuselages of the aircrafts). The areas that are most vulnerable to fractures, such as rivet bores, are frequently subjected to cold expansion, which leads to the generation of residual stress and increase in fatigue life [1]. High cruising speed at high altitudes causes the material to heat up to even 120 °C [2]. This promotes creep of the material and stress relaxation, which directly leads to a decrease in fatigue life. However, aluminium alloys may also operate at higher temperatures, and hence are commonly used for the housings of low-speed coreless generators in wind turbines. During the operation of such generator, at peak power, its temperature may reach ca. 250 °C [3]. Due to the impact of centrifugal forces at increased temperature, the creep process may occur. As a result of such loads, the material changes its properties and its further operation may lead to damage. This change is an effect from a joint impact of the mechanical and thermal loads. Factors such as temperature, strain rate, duration of load, etc. are gaining pivotal significance.

Favourable loading conditions at increased temperature may trigger the process of dynamic recrystallization in the alloy. This significantly affects subsequent mechanical properties of the material, in the case of both monotonic and cyclically varying loads. The recrystallization process of aluminium alloys is constantly undergoing study (e.g., [4,5,6]). Essentially, three types of dynamic recrystallization are distinguished: discontinuous (DDRX)—referring to the nucleation and increase in the size of grains; continuous (CDRX)—referring to the transition of grains with low angle misorientation to grains with high angle misorientation; and geometric (GDRX)—referring to the fragmentation of primary grains [6,7,8]. In the vast majority of aluminium alloys, the dominating mechanism of dynamic recrystallization is CDRX. This process occurs primarily in the case of small primary grains, large precipitation particles, and significant strains [9]. Less-common cases of the DDRX mechanism occurring in aluminium alloys have also been documented [10,11,12].

The process of dynamic recrystallization is significantly affected by temperature, strain level, and strain rate. The fractions with high misorientation angle grow significantly with increase in strain [7]. Even if the percentage of random boundaries is low before deformation, it increases gradually with strain by DRX during deformation [13,14]. Comparison of dynamic recrystallization test results during compression and torsion showed that the strain path does not noticeably alter the DRX kinetics. A DRX process is usually initialized by higher temperature and lower strain rates [15,16,17]. In the case of a constant temperature, the possibility of recrystallization is strongly related to the strain rate [18,19]—a specific strain rate can be observed, below which the DRX does not occur. The non-homogenous distribution of the particles in the vicinity of grain boundaries led to boundary migration caused by the strain [15]. The primary cause of this recrystallization is high dislocation density generated in the close vicinity of these particles [20,21,22]. Additionally, it was observed that complete recrystallization at higher temperature requires lower strain values. A similar correlation was observed in a composite material, where microstructure was obtained with equiaxial grains and high dislocation density [23]. It was found that a lower limit to the processing temperature for the formation of ultra-fine grain structures can be imposed by a low grain boundary mobility [24]. An upper limit can be imposed by grain growth.

Dynamic recrystallization can clearly improve the mechanical properties of the material. This was demonstrated in an alloy obtained by the moderate strain condition imposed by hot extrusion [10]. Here, discontinuous DRX exhibited both high yield stress and elongation. The technology used to manufacture the alloy is also of great significance. The same aluminium alloy obtained by powder metallurgy (PM) may have completely different properties at elevated temperature from the alloy obtained by ingot metallurgy (IM) [13,14]. It was found that a DRX process occurred during deformation in the PM alloy, and static recrystallization occurred in the IM alloy before deformation. Dynamic recrystallization and dynamic recovery (DRV) can also lead to the softening of aluminium alloys at elevated temperature and at different strain rates [19]. However, at lower strain rate, the softening is caused by CDRX, whereas it is caused by DRV at higher strain rate. This stems from the fact that the average misorientation parameter value does not change in the latter case.

A totally recrystallized structure in aluminium alloys can be obtained using different techniques. One of the most popular is equal channel angular extrusion (ECAE) [25]. The structure obtained by this method usually contains grains with an average size of 0.9 μm and relatively low dislocation density. The process of continuous dynamic recrystallization continued in a uniform manner. New grains were formed both along the boundaries of parent grains and inside the existing grains. As was also demonstrated, during the ECAE extrusion, the best results were obtained with constant strain path [26]. The process of microstructural transformations during ECAE can be divided into three stages [27,28]: (1) the formation of classic dislocation structures accompanied by the creation of deformation bands; (2) the growth of large deformation bands leading to the fragmentation of grains; (3) the final growth of new grains. In [29], isothermal rolling after ECAE process was demonstrated to cause an increment in fractions with high misorientation angle, thus further improving the plasticity of the material. The three-stage character of the microstructural transformations was also confirmed [30] for alloy subjected to compression in several directions. Frequently, the process of high-pressure torsion (HPT) is used to obtain a recrystallized structure in different alloys [31,32,33,34]. It brings about additional interesting observations related to the behaviour of the material’s microstructure. The cited papers irrefutably prove that severe plastic deformation (SPD) can not only cause grain refinement, but also accelerates the mass transfer process [33]. In order to analyse such states, information is required regarding, for example, the concentration of a second component in solution, activation enthalpy of bulk tracer diffusion, etc. The HPT technique can lead to the formation of nanostructures which are further from the equilibrium state than the initial coarse-grained material [32].

This paper is focused on the effects connected to the influence of dynamic recrystallization on monotonic and cyclic properties of 2024 aluminium alloy. The possibility of dynamic recrystallization occurring in conditions of monotonic tension and creep tests at the temperatures of 100 °C, 200 °C, and 300 °C was analysed. A constant low strain rate of 0.0015/s was used for this purpose. The creep process at the increased temperatures and constant force was continued until two various degrees of strain were obtained. The material with pre-deformation was subjected to a metallographic analysis, monotonic tensile tests, and low-cycle fatigue. The results of the analysis were compared to the results obtained for as-extruded material.

## 2. Materials and Methods

**Figure 1:**
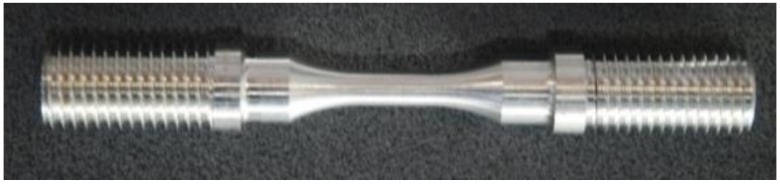
Sample used in monotonic tensile and creep tests.

**Figure 2:**
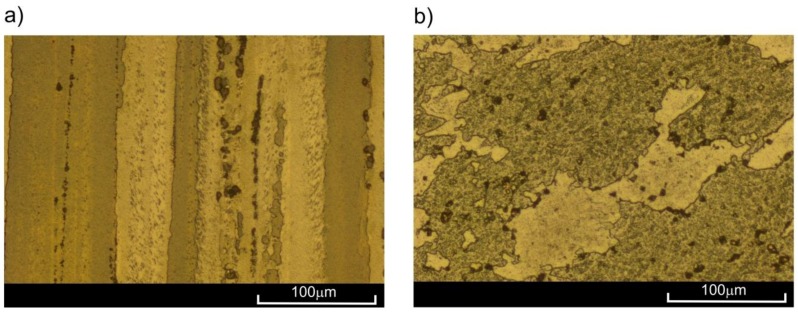
Structure of as-extruded 2024 aluminium alloy in: (**a**) longitudinal; (**b**) transverse cross-section.

The EN AW-2024 aluminium alloy was used in this investigation. The samples (Figure 1) were made from extruded rods with a length of 3 m and diameter of 16 mm. The chemical composition is presented in Table 1. The gauge diameter of the sample was 6.5 mm, and the gauge length was 13 mm. The total length of the sample and the length of the screwed parts were 126 mm and 31 mm, respectively. The structure of the analysed alloy, clearly directed due to the extrusion process, is presented in Figure 2. Keller’s reagent (1.5% HCl + 1% HF + 2.5% HNO_3_ + 95% H_2_O) was used in the etching process.

The samples used in the monotonic tension and creep tests were identical in shape and size. The fastened part of the samples was screwed, as required by the creep testing machine. The monotonic tension and creep tests were conducted using the Kappa 100 SS creep testing machine manufactured by Zwick/Roell (Ulm, Germany). The machine can be used to perform tests of creep, creep rupture, monotonic tension, and stress relaxation. The creep testing machine was controlled with the specialized software testXpert II v3.6.

For analysis at increased temperature, a three-zone furnace manufactured by Maytec Mess und Regeltechnik GmbH (Singen, Germany) was used, with temperature range of up to 900 °C, with a universal Zwick/Roell controller. The furnace is equipped with six thermocouples, three of which are installed on furnace walls inside its working space. The other three thermocouples were mounted on the sample. This enabled recording of the temperature in three different places on the sample as well as the temperature of the furnace. The sample was placed inside the furnace using special high-temperature (up to 1200 °C) strings.

A specifically designed and built device was used to measure the strains [36]. This device allows the sample strain to be measured using any device (e.g., extensometer) that can be used for strain measurement at room temperature. The Epsilon 3542050M-50-ST axial extensometer (Epsilon Technology Corp., Jackson, WY, USA) with varying gauge length of 25/50 mm and range of +25 mm and −5 mm was used in cooperation with the aforementioned device.

In fatigue tests, the servo-hydraulic Material Testing System (MTS) 322 with range of axial force of ±50 kN was used. The strain measurements were taken using an Instron 2620-601 dynamic extensometer (Instron, Norwood, MA, USA) with gauge length of 12.5 mm and range of ±5 mm. The fatigue tests were conducted at room temperature (20 °C) with frequency of *f* = 0.2 Hz and cycle asymmetry coefficient of *S* = ε_min_/ε_max_ = −1 [37]. The samples used in the fatigue tests were different from those used in the creep and tensile tests only in their lack of a thread at the gripped section.

## 3. Results and Discussion

### 3.1. Tests of Monotonic Tensile, Creep-Rupture, and Creep

In tests of monotonic tension and creep at increased temperatures, samples were heated to different temperatures at various rates, as presented in Table 2. Set temperatures were achieved with a precision of ±2 °C. It should be noted that before the start of the test, the samples remained in the furnace until reaching the set temperature. The heating rate and period of sample remaining in the furnace ensured that the required temperature was achieved in the whole gauge area. It must be noted that the data in Table 2 only describe the heating history of the sample in the furnace. The heating rates remained in accordance with the manufacturer’s recommendations.

**Figure 3:**
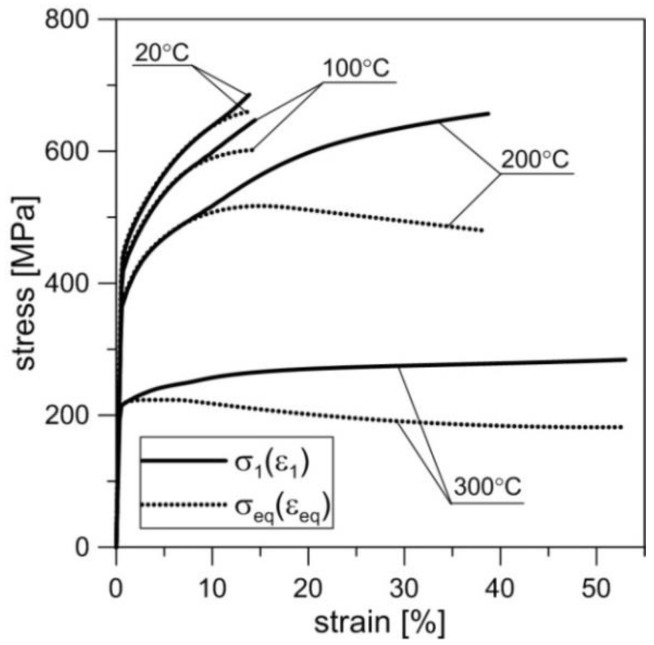
True stress–strain curves of monotonic tension *σ*_1_ = *σ*_1_(*ε*_1_) and *σ*_eq_ = *σ*_eq_(*ε*_eq_) of 2024 aluminium alloy obtained at various temperatures for as-extruded material.

In order to determine the basic mechanical properties of the material at elevated temperature (100 °C, 200 °C, 300 °C) as well as at room temperature (20 °C), the monotonic tensile tests were performed on a series on three samples at each temperature, in accordance with [38,39]. The strain rate of gauge length in these tests was 0.0015/s. The results in the form of true tension curves (*σ*_1_,*ε*_1_) and (*σ*_eq_,*ε*_eq_) are presented in Figure 3. These curves were obtained through numeric calculations using the finite element method including experimental correlations between the force and elongation of the gauge length. The applied procedure was identical to the one presented in the paper by Derpeński and Seweryn [40], which allowed for the determination of maximal principal stress and strain *σ*_1_ and *ε*_1_ as well as equivalent stress and strain *σ*_eq_ and *ε*_eq_. The MSC.Marc software (release 2010) package was used for these simulations. The elastic–plastic material model with isotropic hardening and the Huber–von Mises yield criterion was applied in this procedure. The hardening curve until neck formation was taken directly from the results of the experiment. The remaining part of the curve was determined on the basis of iterative calculations accounting for the necking effect. A nominal hardening curve was initially assumed, followed by load values and their corresponding displacement values being determined and then compared to the values obtained in the experiment. Next, the obtained curve was corrected and iterations progressed until the shape of the load–displacement curve from numerical simulations fitted the actual curve. This enabled the determination of both principal stress and strain (*σ*_1_,*ε*_1_) as well as equivalent stress and strain (*σ*_eq_,*ε*_eq_) according to the Huber–von Mises hypothesis. In Figure 3, two different true stress–strain curves *σ*_1_(*ε*_1_) and *σ*_eq_(*ε*_eq_) are shown. The distributions of stress (or strain) *σ*_1_(*ε*_1_) and *σ*_eq_(*ε*_eq_) prove that the principal stress (or principal strain) in the axis of the sample, in the moment of rupturing, exceeded the equivalent stress (equivalent strain). This correlates closely with the character of the surface of the fractures—the number and shape of the remnants of pores. This serves as the basis for the description of failure mechanism during monotonic tensile and creep tests. The values of basic parameters such as Young’s modulus, yield stress *σ*_y_, ultimate tensile strength *σ*_u_, maximal strain *ε*_u_ corresponding to *σ*_u_, and nominal strain at break *ε*_B_ are presented in Table 3 [41]. It must be noted that the values *σ*_c_ and *ε*_c_ correspond to values of principal stress and strain *σ*_1_ and *ε*_1_ obtained based on true tension curves.

As the temperature increased, the values of the basic mechanical parameters of analysed alloy, such as Young modulus *E*, yield stress *σ*_y_, and ultimate tensile strength *σ*_u_, decreased. This decrease was relatively small at 100 °C and very significant at 300 °C. At the same time, the percentage elongation *ε*_u_, corresponding to *σ*_u_, decreased with the increase in the temperature, whereas percentage elongation at break *ε*_B_—increased. This indicates the increase in ductility of the material from the moment of strain localization and the formation of a neck up to rupture.

**Figure 4:**
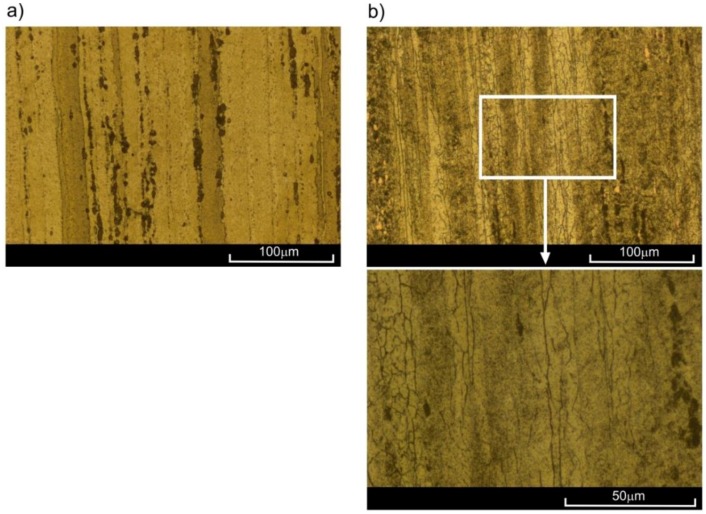
Microstructure of 2024 aluminium alloy subjected to tension at temperatures of (**a**) 200 °C, (**b**) 300 °C, and cooling at ambient temperature.

The observations of the microstructure of the sample ruptured at the temperatures of 20 °C, 100 °C, and 200 °C (Figure 4a) did not present significant changes in comparison to as-extruded material (Figure 2). Let us note that all samples for microstructure observation were taken from the area just below the surface of the fracture in the neighbourhood of the sample axis. However, in the case of samples ruptured at 300 °C, clear changes in microstructure were visible (Figure 4b). As a result of the strain at high temperature, fine equiaxial grains appeared, indicating the occurrence of dynamic recrystallization. New grains appeared mostly on the boundaries of large primary grains, deformed in the direction of loading. The short heating period at elevated temperature did not allow for full recrystallization of the material.

The paper by Tomczyk et al. [41] presents a detailed description of the scanning electron microscope (SEM) fractures of samples obtained by monotonic tension at room and elevated temperatures. The crack initiation process seemed to occur at every temperature at the axis of the sample. The fracture surface had a bi-planar nature, with the central part being a plane perpendicular to the axis. The numerical calculations clearly indicate that the principal stress *σ*_1_ or principal strain *ε*_1_ were dominating on this plane. The initiation of damage usually occurred on the boundary of the coarse precipitation and matrix or on the grain boundaries. The increase in the load led to the deformation of thus-created pores in the direction of the stress *σ*_1_. The neighbouring pores created larger voids. At higher temperatures, the remains of the pores were more elongated towards the sample axis. The share of the area described in the whole cross-section increased with increasing temperature. The second of the mentioned planes connected the central area with the outer surface of the sample and rested at an angle of about 45° to the sample axis, with the maximal shear stress dominating in the area. The fracture process occurred much faster and consisted of the sudden rupturing of bridges between the pores. In the case of relatively low temperature (20 °C, 100 °C), this surface was dominant.

**Figure 5:**
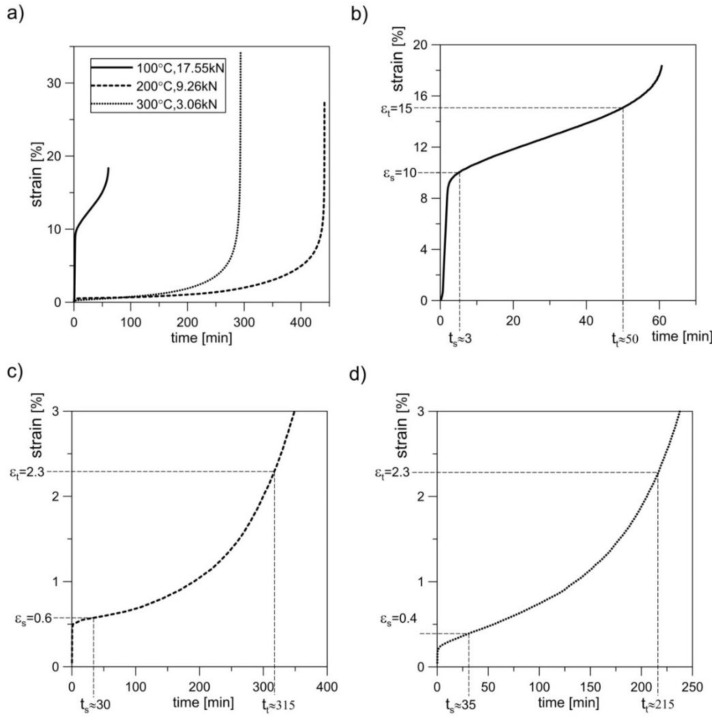
(**a**) Creep-rupture curves of 2024 aluminium alloy obtained in various temperatures and estimated values of time of creep before strain *ε*_s_ and *ε*_t_ for: (**b**) 100 °C, 17.55 kN; (**c**) 200 °C, 9.26 kN; (**d**) 300 °C, 3.06 kN.

**Figure 6:**
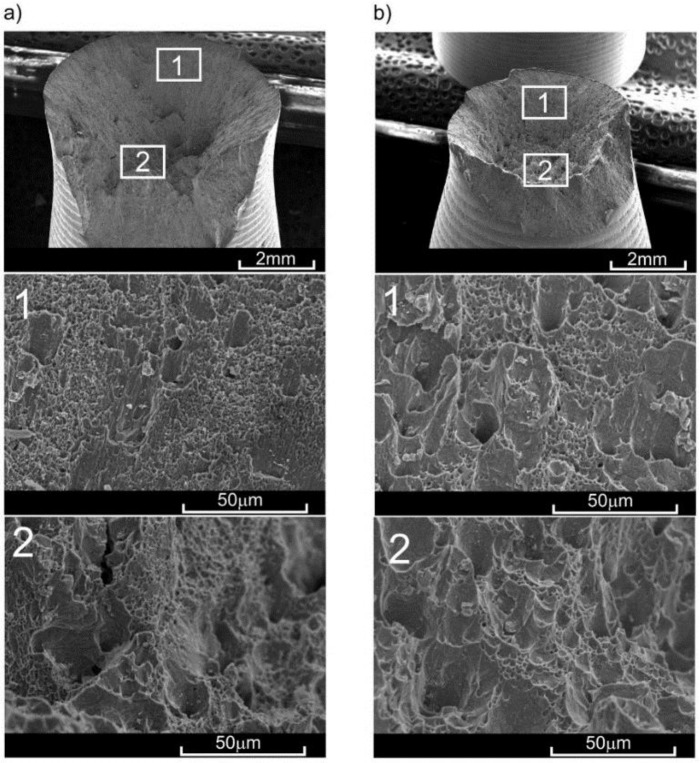
View of fractures obtained in (**a**) monotonic tensile test and (**b**) creep test at a temperature of 200 °C for as-extruded material.

The results obtained from the monotonic tensile tests allowed for the establishment of loads in the creep tests at various elevated temperatures. At 100 °C, the constant force of 17.55 kN was selected, which constituted 1.27 of *σ*_y_ at that temperature. At temperatures of 200 °C and 300 °C, these forces were 9.26 kN (0.75*σ*_y_) and 3.06 kN (0.45*σ*_y_), respectively. The creep tests were performed in accordance with [43] at a loading speed of 0.0015/s. In order to remove any clearances in the grip-pull rod sample system, a preload of up to 50 N was applied prior to the application of proper force. The analysis was conducted on a series of four samples at each temperature, and then the results in the form of creep curves were averaged by time (Figure 5). The character of sample fractures after the creep rupture at various temperatures confirmed the increase in material ductility with the increase in temperature [42]. The character of these fractures was similar to the fractures obtained in monotonic tensile tests (Figure 6). However, the share of surface perpendicular to the sample axis was notably higher in the case of creep.

**Figure 7:**
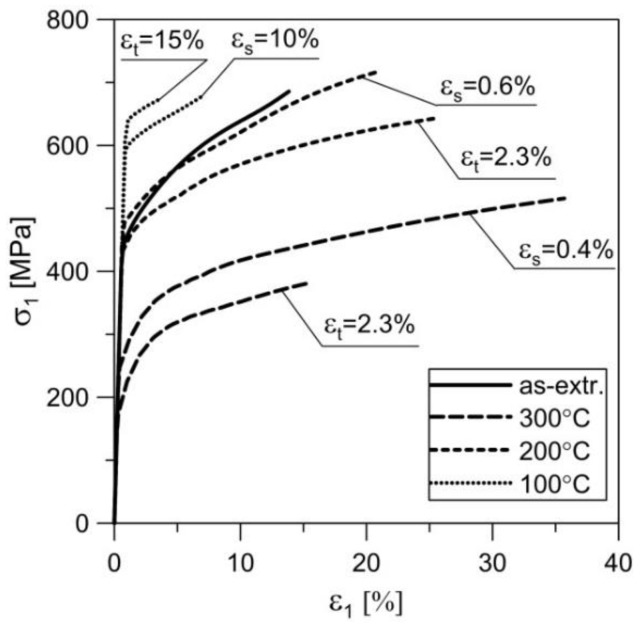
True curves of monotonic tension of 2024 aluminium alloy obtained for samples subjected to creep pre-deformation at various temperatures and with varying degrees of strain compared with tension curve for as-extruded material.

**Figure 8:**
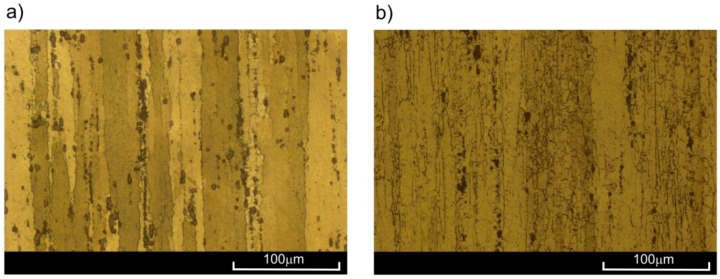
Microstructure of 2024 aluminium alloy subjected to creep at 200 °C until strain of: (**a**) *ε*_s_ = 0.6%; (**b**) *ε*_t_ = 2.3%, cooled at ambient temperature.

**Figure 9:**
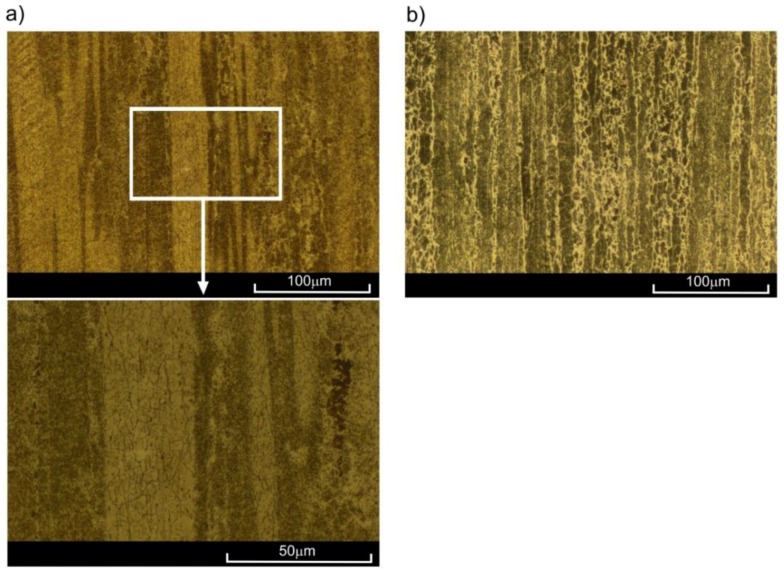
Microstructure of 2024 aluminium alloy subjected to creep at 300 °C for: (**a**) *ε*_s_ = 0.4%; (**b**) *ε*_t_ = 2.3%, cooled at ambient temperature.

The strain level for creep pre-deformation was determined based on the average creep curves. Two different strain levels were selected. The idea was that the smaller deformation *ε*_s_ corresponds to the beginning of the second creep stage. It is worth noting that in the case of creep curves at 200 °C and 300 °C, the second stage was not “perfectly” stable. The value of *ε*_s_ was therefore assumed immediately after the rapid decrease in creep rate (i.e., after reaching the given constant loading). The choice of larger deformation (*ε*_t_) was encouraged by the need to achieve a specific strain close to the end of the secondary creep and the beginning of the tertiary creep. The values of loads during pre-deformation at elevated temperatures were identical to those of creep-rupture tests. At 100 °C, this load equalled 17.55 kN, at 200 °C—9.26 kN, and at 300 °C—3.06 kN. The pre-deformation levels were as follows: for 100 °C—*ε*_s_ = 10%, *ε*_t_ = 15%; for 200 °C—*ε*_s_ = 0.6%, *ε*_t_ = 2.3%; for 300 °C—*ε*_s_ = 0.4%, *ε*_t_ = 2.3%. The process of preliminary creep was conducted in one series of samples at given temperature and given load until *ε*_s_ was obtained, whereas in the other series it was conducted until *ε*_t_ was obtained. The period of sample creep until obtaining *ε*_s_ and *ε*_t_ strains was *t*_s_ and *t*_t_, respectively (Figure 5). The unloading of the sample gauge after reaching the set strain levels took place at a speed of 0.003/s. Cooling of the samples was conducted in open air at room temperature. The strain values *ε*_s_ and *ε*_t_ varied depending on the temperature at which the tests were conducted. Ultimately, two series of pre-deformed samples were obtained per set temperature with each series containing four samples. Three samples from every series were subjected to monotonic tensile tests in order to determine basic mechanical properties [41,42]. The values of these parameters are presented in Table 4. In Figure 7, true curves of monotonic tension *σ*_1_ = *σ*_1_(*ε*_1_) are presented as obtained at room temperature for samples previously subjected to pre-straining at various temperatures and for two different strain levels. The last sample of each series was used for performing sections in order to observe the evolution of microstructure (Figure 8 and Figure 9).

The microstructure of the 2024 aluminium alloy subjected to creep at temperature of 200 °C until strain *ε*_s_ = 0.6% was reached barely differed from the initial structure. Only single equiaxial grains could be observed, appearing mostly on the boundaries of parent grains in the vicinity of clusters of precipitation (Figure 8a). In these conditions, the advanced processes of recrystallization did not occur, which was confirmed by the results of the monotonic tensile tests—the curve *ε*_s_ = 0.6% (200 °C) ran similarly to the curve of the input material (Figure 7). However, a very different picture was presented by the microstructure of alloy subjected to creep at 200 °C with a strain of *ε*_t_ = 2.3%. Multiple fine equiaxial grains are visible in Figure 8b, with their layout corresponding to that of the preliminary grains. In conclusion, dynamic recrystallization (DRX) occurred in this case, which also led to the increase in plasticity (curve *ε*_t_ = 2.3% in Figure 7). Both the appearance of the recrystallized grains throughout the whole volume of parent grains with no clear areas of nucleation and growth, and the fact that the analysed aluminium alloy is characterized by high stacking-fault energy (SFE) [8] indicate that continuous dynamic recrystallization (CDRX) occurred. The significant differences in the structure of alloy pre-deformed at 200 °C with various degrees of strain may also have been a result of the time of creep. At *ε*_s_ = 0.6%, both the strain level and time may have been insufficient for dynamic recrystallization to occur. Similar conclusions can be drawn by analysing results obtained for the alloy pre-deformed at 300 °C with strain of *ε*_s_ = 0.4%. Numerous lines of fine equiaxial grains could be observed in the alloy structure (Figure 9a). The share of the recrystallized structure was greater than in the sample pre-deformed at 200 °C, as confirmed by the increase in plasticity (Figure 7—curve *ε*_s_ = 0.4%, 300 °C). The increase in the degree of strain at 300 °C combined with longer period of creep led to the increase in the range of dynamic recrystallization—the recrystallized structure was visible in a large area of the analysed microstructure.

Creep tests at 200 °C and 300 °C were performed with a force lower than that corresponding with the yield stress, whereas they were performed with greater force at 100 °C. Hence, in the latter case, the material properties after creep pre-deformation were mostly affected by the process of hardening connected to exceeded yield stress and relaxation of the sample. No evidence was found here that would indicate the process of recrystallization. Despite significant strains, the temperature of 100 °C was too low to initiate the process, hence the significant increase in the offset yield stress and the simultaneous decrease in elongation (Figure 7, Table 4).

**Figure 10:**
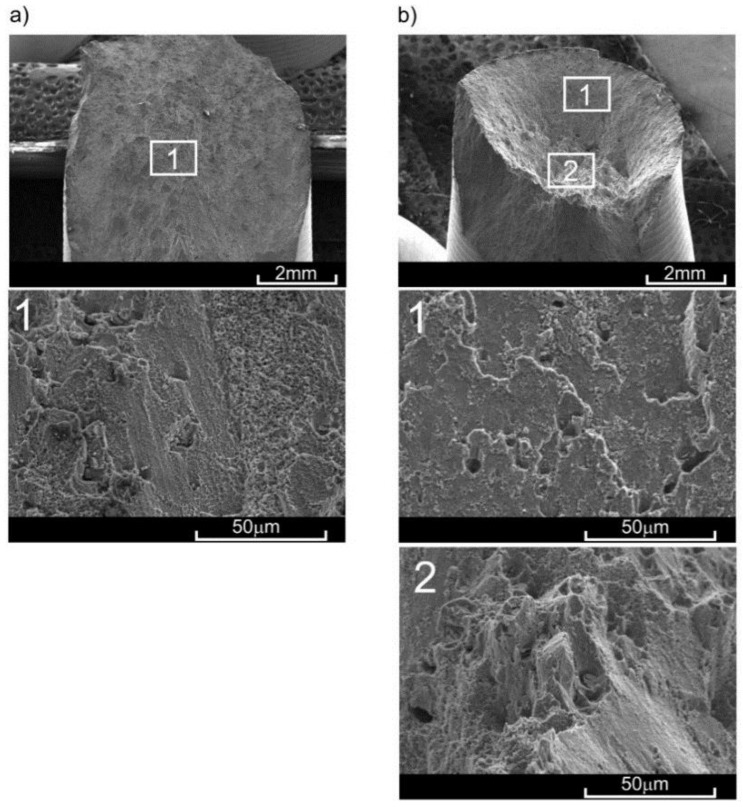
Surface of fractures obtained in monotonic tensile test at ambient temperature with pre-deformation for: (**a**) *ε*_s_ = 10% at 100 °C; (**b**) *ε*_s_ = 0.4% at 300 °C.

The character of the fracture surface in samples pre-deformed at 200 °C was affected mostly by preliminary strain and the time for which the sample remained at increased temperature [42]. For the preliminary strain of *ε*_s_ = 0.6%, hardening of material occurred (with respect to as-extruded material), followed by subsequent softening. During the pre-deformation of *ε*_s_ = 0.6%, no significant strains occurred—only a few voids appeared, and the remnants after their clear shearing were spherical rather than elongated in the shape [41]. Several precipitations of intermetallic phases were observed. The lack of great numbers of voids after preliminary strain resulted in an increase in yield stress in the process of monotonic tension. However, the subsequent growth of these voids caused the material to be incapable of carrying shear stress, causing them to be cut easily and rapidly which could result in the reduction of ultimate tensile strength. It should be highlighted that the time during which the material remains at the elevated temperature has a great impact, also significantly affecting the values of the basic mechanical properties (e.g., [44,45,46]). In the case of pre-deformation at 200 °C, this time varied significantly for *ε*_s_ = 0.6% (ca. 30 min) and *ε*_t_ = 2.3% (ca. 300 min). In the case of larger pre-strain (*ε*_t_ = 2.3), the pores were joining and clearly deforming towards the direction of axial load in the central area of the cross section. After unloading, the pores which had not ruptured reassumed spherical shape and were ruptured during tension at room temperature (i.e., with smaller strain). The process of rupturing in the central area of the sample preceded ultimate shearing of the material. The fractures obtained in the samples with lower pre-deformation (*ε*_s_) at temperatures of 100 °C and 200 °C were characterized by a large number of remnants (dimples) of small-sized pores (Figure 10).

In the case of greater pre-deformation (*ε*_t_) at the same temperatures, small dimples covered much less surface in favour of large dimples. A similar effect was obtained by Lin et al. [47] by increasing temperature with the same stress or increasing stress at the same temperature during pre-deformation of the 2024 alloy. However, a similar effect of pore growth due to the increase in stress during creep rupture tests on the 2124 alloy at 260 °C was obtained by Li et al. [48]. The character of the fracture in the case of samples pre-deformed at 300 °C did not indicate any significant differences in terms of the value of this pre-deformation. The fracture initiation plane, perpendicular to the sample axis, was clearly marked, as was the shearing plane (Figure 10b). The share of the former in the total surface of the fracture was significantly lower than in the case of, for example, tension at 300 °C.

### 3.2. Low-Cycle Fatigue (LCF) Tests

Low-cycle fatigue tests were conducted on as-extruded material and material with pre-deformation at 200 °C and 300 °C. Undamaged (as-extruded) material was chosen for comparison purpose. The material with pre-deformation–due to the possibility of investigating the impact of the DRX process on the cyclic properties of the material.

The fatigue tests were conducted in accordance with the conditions described in the last paragraph of the Section 2. Five degrees of total strain amplitude were selected (i.e., 0.02, 0.01, 0.008, 0.005, 0.0035), and tests on each level were repeated three times. The description of the fatigue life was made using one of the most common models, namely the Manson–Coffin–Basquin model [49,50,51,52]:

(1) $$\varepsilon_{a}=\varepsilon_{\mathrm{ae}}+\varepsilon_{\mathrm{ap}}=\frac{\sigma_{f}^{\prime }}{E}{\left(2N_{f}\right)}^{b}+\varepsilon_{f}^{\prime }{\left(2N_{f}\right)}^{c},$$

where values *ε*_ae_, *ε*_ap_ are the amplitude of elastic and plastic strain respectively; $\sigma_{f}^{\prime },\;\varepsilon_{f}^{\prime }$ are coefficients of fatigue strength and ductility; *b* and *c* are exponents of fatigue strength and ductility; *N*_f_ is the number of cycles to failure.

The cyclic properties of the material allowing for the identification of susceptibility to hardening or softening are described with the following formula (e.g., [53,54]):

(2) $$\sigma_{a}=K^{\prime }{\left(\varepsilon_{\mathrm{ap}}\right)}^{n^{\prime }},$$

where *σ*_a_ is the stress amplitude; $n^{\prime }$ is the exponent of plastic hardening; $K^{\prime }$ is a material parameter.

**Figure 11:**
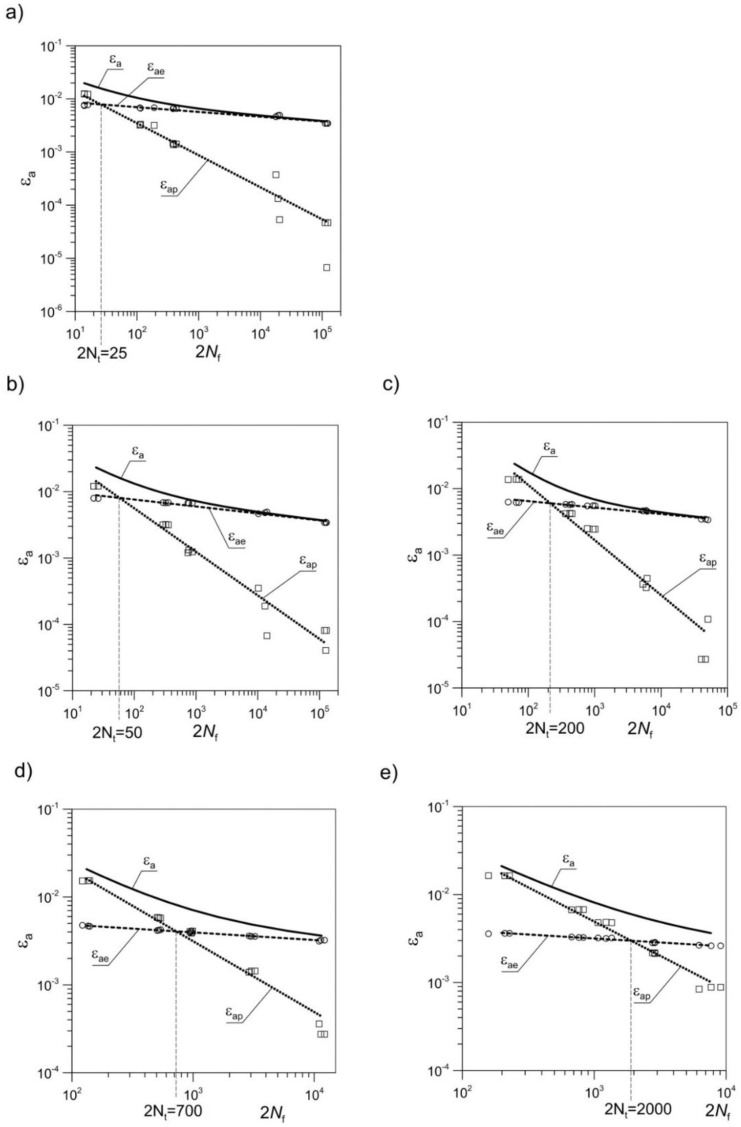
Fatigue life curves *ε*_a_(2*N*_f_) in logarithmic scale at ambient temperature obtained for EN AW-2024 aluminium alloy for (**a**) as-extruded material, and with pre-deformation at temperatures of (**b**) 200 °C (*ε*_s_ = 0.6%), (**c**) 200 °C (*ε*_t_ = 2.3%), (**d**) 300 °C (*ε*_s_ = 0.4%), and (**e**) 300 °C (*ε*_t_ = 2.3%).

In Figure 11, curves of fatigue life obtained for as-extruded material are compared with those obtained for material with pre-deformation at 200 °C and 300 °C. The values of the data from LCF tests, on the basis of which the diagrams in Figure 11 were built, are presented in Table 5. The number of cycles to failure is denoted by *N*_f_, the amplitude of the strain control variable by *ε*_a_, and the amplitude of the stress by *σ*_a_. Furthermore, only the cases in which a recrystallization process occurred were taken into account. As a result of pre-deformation, a significant increase in fatigue life occurred in the areas dominated by plastic deformation. This was particularly noticeable in the cases where the process of dynamic recrystallization was most efficient, namely for the temperature of 300 °C and significant strains of *ε*_t_ = 2.3% (Figure 11). At the same time, the fatigue life decreased in the areas dominated by elastic strains. For instance, the fatigue life for strain of *ε*_a_ = 0.02 for material pre-deformed at *ε*_s_ = 0.4% and temperature of 300 °C increased from 7 to 66 cycles as compared with the as-extruded one, whereas in the case of material pre-deformed at *ε*_t_ = 2.3% and the same temperature, fatigue life increased to 99 cycles [41]. In the case of the material pre-deformed at *ε*_t_ = 2.3% at a temperature of 200 °C, the fatigue life increased to 31 cycles. The transition number of cycles 2*N*_t_ increased as well (Figure 11).

**Figure 12:**
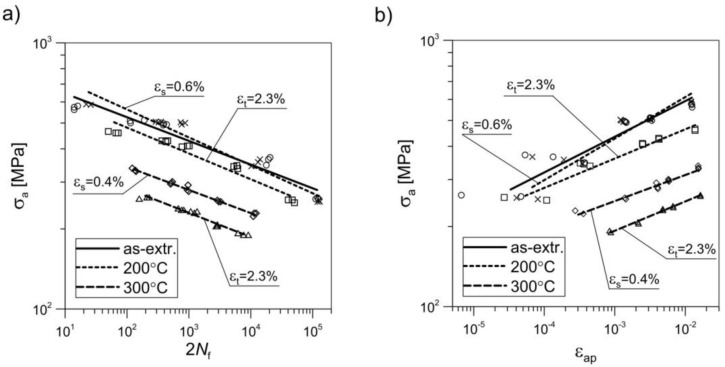
(**a**) Fatigue life curves *σ*_a_(2*N*_f_) in logarithmic scale and (**b**) curves of cyclic strain *σ*_a_(*ε*_ap_) at ambient temperature as obtained for samples of 2024 aluminium alloy with pre-deformation at various temperatures and with varying degrees of strain.

With the constant value of control variable *ε*_a_, the increase in fatigue life was observed to come at the cost of decline in fatigue strength (Figure 12a). This was again particularly visible in the cases where recrystallization covered the largest number of grains (i.e., for pre-deformation at 300 °C). This applied to both low-cycle and high-cycle loads. In the case of control variable *ε*_a_ = 0.02 and material pre-deformed at *ε*_s_ = 0.4% and temperature of 300 °C, strength decreased from 571 MPa to 333 MPa (by ca. 40%), whereas it decreased to 262 MPa (by ca. 54%) in the case of material pre-deformed at *ε*_t_ = 2.3% at the same temperature [41]. The decrease in the fatigue life for the material pre-deformed at 200 °C (*ε*_t_ = 2.3%) with the control variable of *ε*_a_ = 0.0035 proceeded while retaining the same level of strength (Figure 12a).

**Figure 13:**
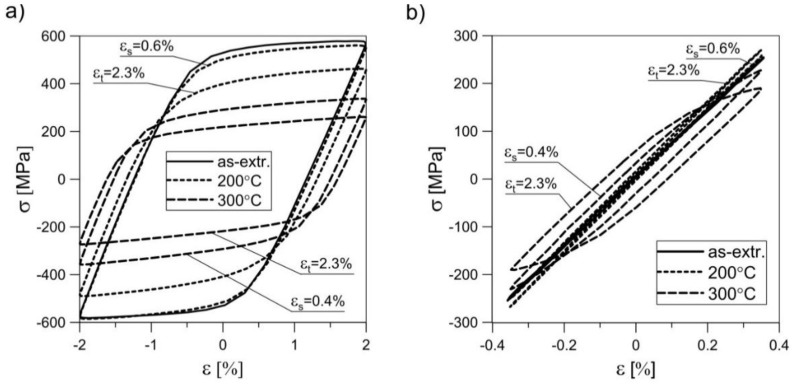
Stabilized hysteresis loops obtained for EN AW-2024 aluminium alloy subjected to creep pre-deformation at various temperatures and with varying degrees of strain: (**a**) *ε*_a_ = 0.02; (**b**) *ε*_a_ = 0.0035.

Figure 12b depicts the change in the cyclic plasticity of the pre-deformed material in comparison with the as-extruded material. The dynamic recrystallization led to improved plasticity and an increase of the plastic flow phase during cyclic loading (Figure 13).

However, this came at the cost of a reduction in the fatigue strength. As a result, the material in which recrystallization proved to be most efficient at low loading amplitudes (0.0035) had a clear wide hysteresis loop. The loops of the less-recrystallized material were very narrow—there were no plastic deformations detected. It should be noted that the pre-deformation at *ε*_s_ = 0.6% at 200 °C only slightly affected the cyclic properties of the material as compared to the as-extruded material. This applied to both small and large values of the control variable *ε*_a_. This is particularly interesting considering the fact that the basic mechanical parameters of the material changed significantly (see Table 4). A different situation could be observed for the material with pre-deformation of *ε*_t_ = 2.3% at 200 °C. Here one may notice a significant change in both cyclic and monotonic behaviour.

**Figure 14:**
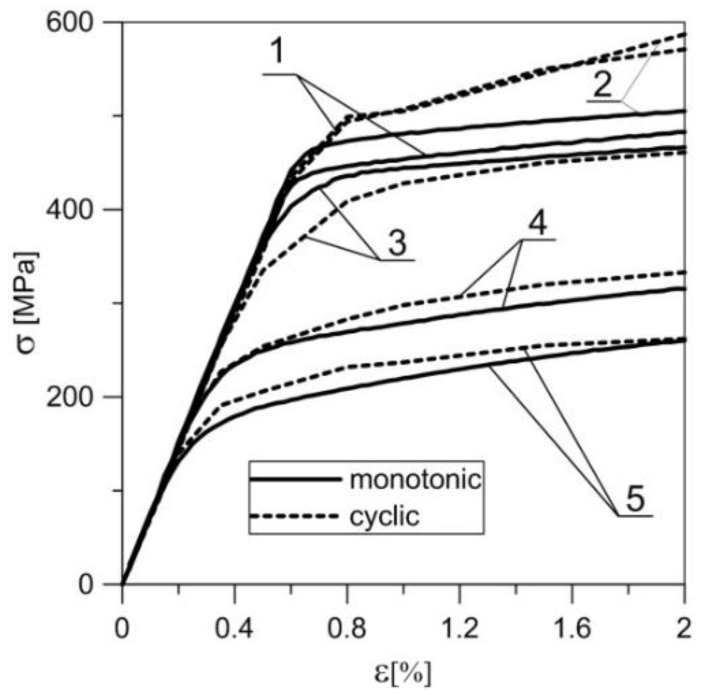
Curves of monotonic and cyclic tension at ambient temperature obtained for EN AW-2024 aluminium alloy samples pre-deformed at various temperatures and with varying degrees of strain: **1**—as-extruded; **2**—200 °C, ε_s_ = 0.6%; **3**—200 °C, ε_t_ = 2.3%; **4**—300 °C, ε_s_ = 0.4%; **5**—300 °C, ε_t_ = 2.3%.

The process of dynamic recrystallization also led to a decrease in the material’s cyclic hardening capability (Figure 14). As-extruded material indicated clear susceptibility to high (ca. 20%) cyclic hardening [41]. As a result of the pre-deformation at the temperature of 300 °C, on both levels of strain, the cyclic strengthening was insignificant. In the range of strains between 0% and 2%, the material was practically cyclically stable, whereas pre-deformation at the temperature of 200 °C and strain level of *ε*_t_ = 2.3% led to cyclic softening of the material. Metallographic sections of the samples subjected to fatigue tests were also analysed. However, these did not demonstrate any significant differences in comparison with the sections of the samples previously subjected to pre-strain at increased temperature.

This paper presents results of our study on the influence of preliminary creep at elevated temperature on the monotonic and cyclic properties of 2024 aluminium alloy. In the case of low temperature (100 °C) and high strain value, these properties were determined to be affected mostly by mechanical hardening of the material. In the case of higher temperatures (200 °C, 300 °C), the possibility of continuous dynamic recrystallization gained significance. This possibility occurred even at low loading speeds (i.e., 0.0015/s), although in the monotonic tension process only at the temperature of 300 °C. In the case of creep at 200 °C, the recrystallization process occurred only in cases of loading until a greater level of pre-strain *ε*_t_ = 2.3% was achieved. In the case of creep at 300 °C, this process occurred for preliminary strain of both *ε*_s_ = 0.4% and *ε*_t_ = 2.3%. However, in the latter case, the process covered a noticeably greater number of grains.

## 4. Conclusions

Creep pre-deformation at various temperatures allows for the shaping of both monotonic and cyclic properties of the material. The selection of proper parameters of this pre-deformation (e.g., temperature, loading force, or strain level) allows for the improvement of mechanical parameters. This relates to the effectiveness of dynamic recrystallization. The effect of dynamic recrystallization can be summarized as follows:

1. In the case of the creep pre-deformation at 300 °C, the increase of the value of pre-deformation could also be observed to worsen the basic mechanical parameters in comparison with as-extruded material. However, in the case of the pre-deformation at 200 °C and low values of preliminary strain, the increased yield stress was obtained accompanied by a decrease in the ultimate tensile strength. On the other hand, greater preliminary strains led to the worsening of both yield and ultimate tensile strength.
2. A significant increase in the strain-controlled fatigue life was observed in the area dominated by plastic deformation in the samples with creep pre-deformation at 300 °C, where clear dynamic recrystallization occurred. This applied to the samples pre-deformed to both values of strain (*ε*_s_ = 0.4% and *ε*_t_ = 2.3%), although the improvement was more significant for *ε*_t_.
3. The inverse situation could be observed in the area dominated by elastic strains. In this case, the fatigue life decreased in comparison with as-extruded material.
4. The improvement of the fatigue life was determined to take place at the cost of the decline of its fatigue strength at constant value of the strain-control variable. Such regularity was most visible in the material pre-deformed at the temperature of 300 °C, and was much less visible at 200 °C.

During creep at elevated temperatures, a continuous dynamic recrystallization (CDRX) occurs. The degree of recrystallization increases with the increase in temperature and strain level, which significantly affects the plasticity of the material.

## Figures and Tables

**Table 1:** Chemical composition of analysed alloy [35].

Component	Si	Fe	Cu	Mn	Mg	Cr	Zn	Ti
**Amount (%)**	0.13	0.25	4.4	0.62	1.7	0.01	0.08	0.05

**Table 2:** Sample heating rates used in analyses and estimated time of heating to obtain set temperatures.

Set Temperature (°C)	Heating Rate (°C/min)	Estimated Time of Heating (min)
100	3	170 (±5)
200	5	120 (±3)
300	8	100 (±2)

**Table 3:** Values of basic strength parameters of 2024 aluminium alloy and value of critical stress and strain *σ*_c_, *ε*_c_ [42].

*T* (°C)	*E* (GPa)	*σ*_y_ (MPa)	*σ*_u_ (MPa)	*σ*_c_ (MPa)	*ε*_u_ (%)	*ε*_B_ (%)	*ε*_c_ (%)
20	74	447	580	686	12.1	13.3	13.8
100	71	418	536	647	10.3	12.7	14.5
200	70	372	460	657	9.1	19.8	38.7
300	56	214	219	283	2.1	17.0	52.9

**Table 4:** Values of basic strength parameters of 2024 aluminium alloy and values of critical stress and strain *σ*_c_, *ε*_c_ for materials with varying history of pre-deformation [41].

Pre-Deformation Condition			*E* (GPa)	*σ*_y_ (MPa)	*σ*_u_ (MPa)	*σ*_c_ (MPa)	*ε*_u_ (%)	*ε*_B_ (%)	*ε*_c_ (%)
*T* (°C)	*ε*_s_ (%)	*ε*_t_ (%)							
100	10	-	72	598	610	680	4.6	5.3	7.1
	-	15	72	635	640	673	2.7	2.7	3.5
200	0.6	-	74	475	550	716	7.9	13.9	20.8
	-	2.3	74	443	490	642	4.9	13.1	25.3
300	0.4	-	71	255	363	518	7.1	17.9	36.5
	-	2.3	73	186	316	380	8.7	13.1	15.2

**Table 5:** Low-cycle fatigue (LCF) data obtained for as-extruded material and material with pre-deformation at 200 °C and 300 °C [42].

*ε*_a_	As-Extruded		Pre-Deformed at 200 °C			Pre-Deformed at 300 °C		
	*N*_f_	*σ*_a_ (MPa)	*ε*_s_(*ε*_t_) (%)	*N*_f_	*σ*_a_ (MPa)	*ε*_s_(*ε*_t_) (%)	*N*_f_	*σ*_a_ (MPa)
0.02	7	571	*ε*_s_ = 0.6	12	587	*ε*_s_ = 0.4	66	333
			*ε*_t_ = 2.3	31	461	*ε*_t_ = 2.3	99	262
0.01	69	507	*ε*_s_ = 0.6	162	505	*ε*_s_ = 0.4	261	298
			*ε*_t_ = 2.3	208	428	*ε*_t_ = 2.3	379	237
0.008	202	494	*ε*_s_ = 0.6	395	499	*ε*_s_ = 0.4	483	283
			*ε*_t_ = 2.3	455	409	*ε*_t_ = 2.3	610	232
0.005	9576	361	*ε*_s_ = 0.6	6235	355	*ε*_s_ = 0.4	1528	254
			*ε*_t_ = 2.3	2906	342	*ε*_t_ = 2.3	1420	206
0.0035	58,965	260	*ε*_s_ = 0.6	62,739	254	*ε*_s_ = 0.4	5747	227
			*ε*_t_ = 2.3	22,914	255	*ε*_t_ = 2.3	3836	191
